# A ketogenic diet enhances fluconazole efficacy in murine models of systemic fungal infection

**DOI:** 10.1128/mbio.00649-24

**Published:** 2024-04-15

**Authors:** Julia R. Palmucci, Blake E. Sells, Charles D. Giamberardino, Dena L. Toffaletti, Baodi Dai, Yohannes G. Asfaw, Laura G. Dubois, Zhong Li, Barbara Theriot, Wiley A. Schell, William Hope, Jennifer L. Tenor, John R. Perfect

**Affiliations:** 1 Division of Infectious Diseases, Department of Medicine, Duke University School of Medicine, Durham, North Carolina, USA; 2 Department of Medicine, Washington University in St. Louis, St. Louis, Missouri, USA; 3 Department of Laboratory Animal Resources, Duke University Medical Center, Durham, North Carolina, USA; 4 Duke Proteomics and Metabolomics Core Facility, Duke University, Durham, North Carolina, USA; 5 Department of Pediatrics, Duke University School of Medicine, Durham, North Carolina, USA; 6 Antimicrobial Pharmacodynamics and Therapeutics, University of Liverpool, Liverpool Health Partners, Liverpool, United Kingdom; The University of British Columbia, Vancouver, British Columbia, Canada

**Keywords:** *Cryptococcus*, cryptococcal meningitis, *Candida*, ketogenic diet, fluconazole

## Abstract

Invasive fungal infections are a significant public health concern, with mortality rates ranging from 20% to 85% despite current treatments. Therefore, we examined whether a ketogenic diet could serve as a successful treatment intervention in murine models of *Cryptococcus neoformans* and *Candida albicans* infection in combination with fluconazole—a low-cost, readily available antifungal therapy. The ketogenic diet is a high-fat, low-carbohydrate diet that promotes fatty acid oxidation as an alternative to glycolysis through the production of ketone bodies. In this series of experiments, mice fed a ketogenic diet prior to infection with *C. neoformans* and treated with fluconazole had a significant decrease in fungal burden in both the brain (mean 2.66 ± 0.289 log_10_ reduction) and lung (mean 1.72 ± 0.399 log_10_ reduction) compared to fluconazole treatment on a conventional diet. During *C. albicans* infection, kidney fungal burden of mice in the keto-fluconazole combination group was significantly decreased compared to fluconazole alone (2.37 ± 0.770 log_10_-reduction). Along with higher concentrations of fluconazole in the plasma and brain tissue, fluconazole efficacy was maximized at a significantly lower concentration on a keto diet compared to a conventional diet, indicating a dramatic effect on fluconazole pharmacodynamics. Our findings indicate that a ketogenic diet potentiates the effect of fluconazole at multiple body sites during both *C. neoformans* and *C. albicans* infection and could have practical and promising treatment implications.

**IMPORTANCE:**

Invasive fungal infections cause over 2.5 million deaths per year around the world. Treatments for fungal infections are limited, and there is a significant need to develop strategies to enhance antifungal efficacy, combat antifungal resistance, and mitigate treatment side effects. We determined that a high-fat, low-carbohydrate ketogenic diet significantly potentiated the therapeutic effect of fluconazole, which resulted in a substantial decrease in tissue fungal burden of both *C. neoformans* and *C. albicans* in experimental animal models. We believe this work is the first of its kind to demonstrate that diet can dramatically influence the treatment of fungal infections. These results highlight a novel strategy of antifungal drug enhancement and emphasize the need for future investigation into dietary effects on antifungal drug activity.

## INTRODUCTION

Invasive fungal infections (IFIs) present a critical but often overlooked threat to global human health, causing over 2.5 million deaths annually (1). Incidence of IFIs has continued to increase despite advances in other areas of public health, in part due to an increasing population of immunocompromised patients who are particularly susceptible to these infections. The accompanying high mortality rates for many of these infections led the World Health Organization (WHO) to release a fungal priority pathogens list in 2022 (2). The WHO designated *Cryptococcus neoformans* and *Candida albicans* as the highest-priority fungal pathogens due to their impact on public health and treatment difficulties. Cryptococcal meningitis causes over 147,000 deaths worldwide per year and accounts for nearly 20% of all AIDS-related mortality (1, 3). Candidemia and invasive candidiasis are estimated to cause 995,000 deaths per year, and the growing incidence of antifungal resistance, especially resulting from non-albicans *Candida* species like *Candida auris*, is of serious clinical concern (1, 4).

Dietary interventions have long been used for ailments ranging from food intolerances to epilepsy. However, there is growing interest to adapt the concept to a wider variety of medical disorders. In particular, the ketogenic diet (KD) has gained prominence over the last two decades for its implications in a wide variety of disease states including dementia, cancer, and infectious diseases (5–7). The KD is a high-fat, very low carbohydrate diet originally developed in the 1920s as a treatment for epilepsy (8). While on the diet, carbohydrates are limited to 5% of the daily caloric intake. At least 75% of calories consumed by the individual are from fat, and sufficient protein is provided. The decrease in circulating glucose and the subsequent increase in ketone bodies induces ketosis, a process by which fatty acid oxidation becomes the primary energy acquisition strategy (8, 9). This effect is particularly potent in the brain, where ketone bodies are readily transported across the blood-brain barrier by monocarboxylate transporters, the expression of which directly correlates with blood-ketone levels (10). Since solid tumors exhibit dramatically increased levels of glucose uptake, the effect of a ketogenic diet on brain metabolism has become of great interest to the field of neuro-oncology (11).

Carbohydrates, and more specifically glucose, are important growth substrates for fungal pathogens, and its environmental concentrations can affect melanin formation and hyphal transition (12, 13). In *C. albicans*, glucose promotes stress and azole resistance and positively affects adhesion and biofilm formation (14–16). Clinically, variations in patient blood glucose levels have been shown to affect susceptibility or severity of fungal diseases, with prolonged hyperglycemia as a risk factor for poorer outcomes during IFIs (17). Furthermore, patients with diabetes mellitus are at increased risk of invasive candidiasis, and a growing body of work has identified diabetes as a predisposing disease for cryptococcosis in apparently immunocompetent patients (18–21).

Given the observations that glucose may be a risk factor for infection, we asked whether decreasing the amount of circulating glucose during ketosis, without starving the host of necessary energy, could positively impact disease outcome. Thus, we evaluated the efficacy of a KD with and without fluconazole treatment on tissue fungal burden in murine models of systemic cryptococcosis and candidiasis. We demonstrated an altered pharmacokinetic profile of fluconazole during KD treatment and subsequently identified changes in the fluconazole-dose, fungal burden-response relationship, indicating a significant pharmacodynamic effect. Taken together, our potent *in vivo* combination of ketosis and azole therapy highlights the importance of considering the effects of host nutritional state on optimal antifungal drug efficacy and indicates the potential for consideration of low-cost dietary interventions as a supplemental strategy in the treatment of IFIs.

## RESULTS

### A ketogenic diet potentiates the antifungal effect of fluconazole during *Cryptococcus* infection

We used our delayed treatment model of cryptococcal meningitis to assess *C. neoformans* fungal burden in the brain and lungs of mice fed a conventional diet (CD) or KD in the presence and absence of fluconazole. Half of each study cohort began a KD 10 days prior to infection. Mice were infected intravenously with *C. neoformans*, and each dietary cohort was administered vehicle (VEH) or 80-mg/kg fluconazole intraperitoneally (i.p.) beginning 24 h post-inoculation. Blood-ketone measurements demonstrated that KD-fed mice enter ketosis by 10 days post-dietary intervention, as evidenced by elevated blood-ketone levels, and remained in a ketotic state for the duration of the study (Fig. S1A). Blood-glucose levels were also significantly decreased by day 5 post-inoculation in both KD groups compared to conventionally fed mice (Fig. S1B). Fungal burden was assessed on day 6 post-inoculation.

**Fig 1:**
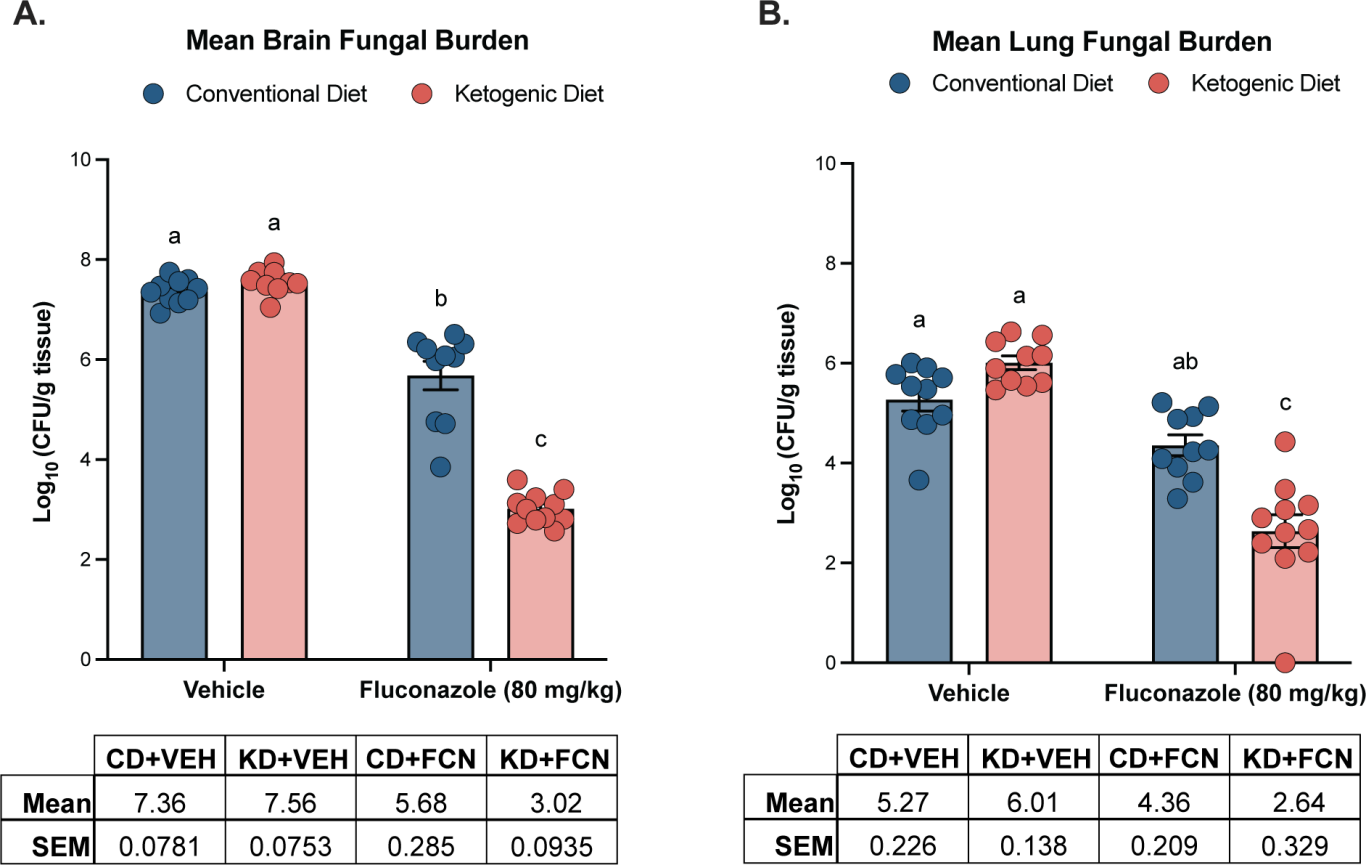
Mice given a KD 10 days prior to inoculation with *C. neoformans* display significantly less brain and lung fungal burden after fluconazole treatment, compared to those fed a CD. (**A**) A 1.78 log_10_ difference in fungal burden was observed between fluconazole and vehicle-treated groups on a CD (*P* < 0.0001), while fluconazole treatment in combination with a KD displayed a 4.24 log_10_ reduction compared to the untreated KD cohort (*P* < 0.0001). While there was no difference between brain fungal burden in untreated KD or CD mice, there was a 2.61 log_10_ decrease between fluconazole-treated dietary groups (*P* < 0.0001). (**B**) In the lung, fluconazole therapy resulted in a 1.46 log_10_ reduction in fungal burden on a CD (*P* < 0.0001) and a 3.28 log_10_ reduction on a KD (*P* < 0.0001) compared to vehicle. While there was no statistical difference between untreated KD and CD lung fungal burden, fluconazole treatment resulted in increased 1.18 log_10_ reduction in fungal burden between CD and KD fluconazole-treated mice (*P* < 0.0001). Mean ± SEM reported for *n* ≥ 10 mice per group of one independent experiment. Two-way analysis of variance with post hoc Bonferroni multiple comparison test was used.

The fungal burden of vehicle-treated mice showed no difference in brain or lung fungal burden (mean log_10_ CFU per gram of tissue) between KD or CD groups (Fig. 1). Fluconazole treatment showed a 1.68 ± 0.296 log_10_ CFU/g reduction in mean brain fungal burden on a CD and a 4.54 ± 0.122 log_10_ CFU/g reduction of the mean brain fungal burden on a KD compared to vehicle controls within each diet (*P* < 0.0001). Notably, there was a 2.66 ± 0.289 log_10_ CFU/g difference in mean brain fungal burden between KD and CD fluconazole-treated groups (KD + fluconazole (FCN) and CD + FCN), indicating enhancement of antifungal activity (*P* < 0.0001). In the lungs, there was a significant reduction in mean fungal burden between vehicle and fluconazole-treated groups of 0.913 ± 0.308 and 3.37 ± 0.370 log_10_ CFU/g on a CD (*P* = 0.0062) and KD (*P* < 0.0001), respectively. Fluconazole antifungal activity also appeared to be potentiated within the lungs as there was a 1.72 ± 0.399 log_10_ CFU/g reduction in mean lung fungal burden between CD + FCN and KD + FCN groups (*P* < 0.0001). The significant difference in fungal burden between KD + FCN and CD + FCN groups in brain and lung tissues was highly replicable. Histological analysis of lung tissue was performed at day 6 post-inoculation. Gross assessment of *Cryptococcus* infiltration supported our CFU quantifications, with fewer yeast cells visible in the fluconazole-treated groups (data not shown).

### A ketogenic diet enhances fluconazole activity against *Candida albicans*

To determine whether the interaction between a KD and fluconazole was specific to *C. neoformans*, we evaluated the combination treatment in a murine model of systemic *Candida albicans* infection. After 10 days on either a KD or CD, mice were infected intravenously with *C. albicans*. Treatment with vehicle or 0.25-mg/kg fluconazole i.p. daily began 24 h post-inoculation. Blood ketone and glucose measurements were taken on the day of dietary intervention, 24 h before infection, and 24 h before euthanasia. Blood ketone measurements were elevated in both KD groups compared to CD mice (Fig. S1C). Blood glucose levels were lower in KD groups compared to CD 1 day before infection. However, the differences were eliminated by day 7 post-inoculation, indicating a possible effect of *Candida* infection on blood glucose levels (Fig. S1D).

**Fig 2:**
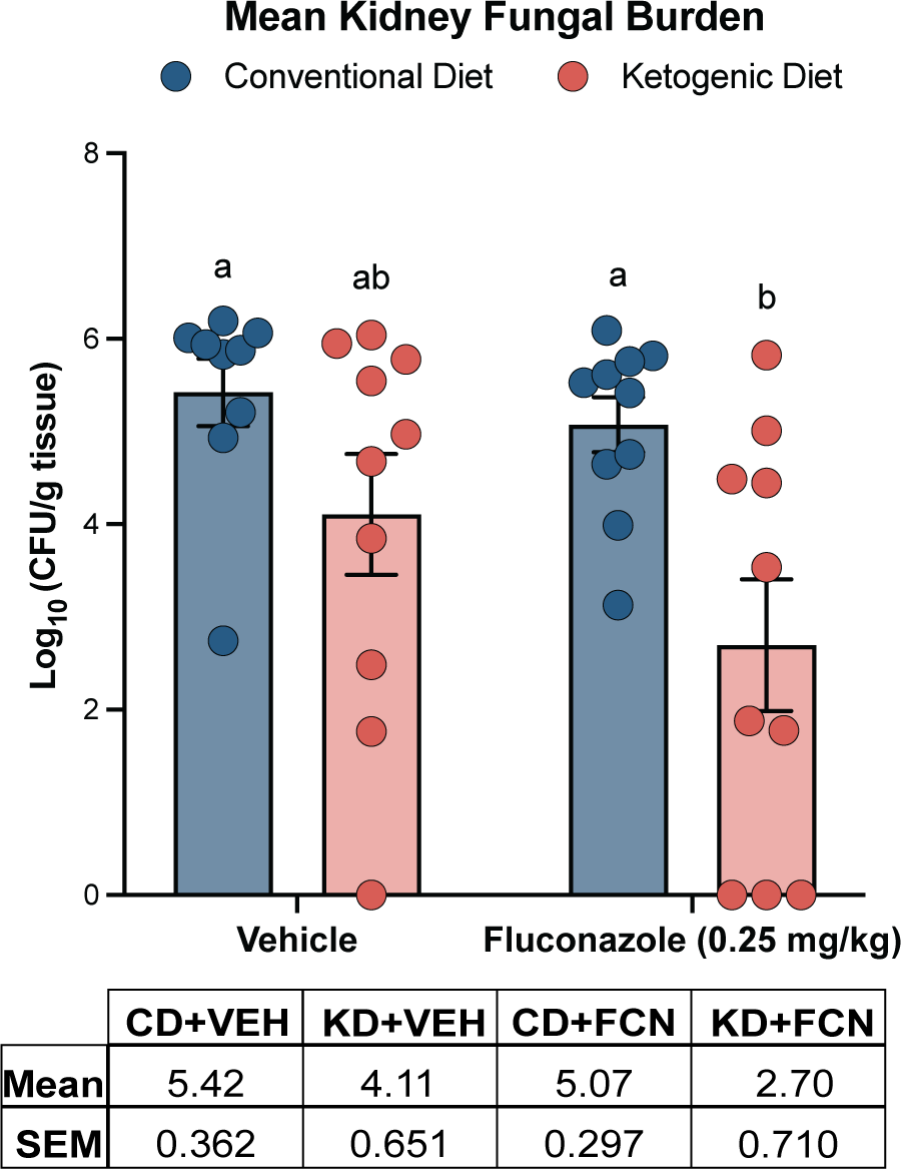
KD-induced potentiation of fluconazole therapy is effective in a *C. albicans* infection model. Fluconazole therapy on a KD resulted in a 2.37 log_10_ reduction in kidney fungal burden compared to a CD (*P* = 0.0078). No difference was observed in kidney fungal burden between untreated CD and KD mice. Mean ± SEM reported for *n* ≥ 9 mice per group of one independent experiment. Two-way analysis of variance with post hoc Bonferroni test was used.

Fungal burden was assessed in the kidney 8 days post-inoculation. There was no difference in fungal burden between KD or CD vehicle-treated groups (KD + VEH and CD + VEH), which was consistent with our observations of lung and brain fungal burden with *C. neoformans* infection (Fig. 2). There was a general trend of reduced fungal burden with fluconazole treatment within dietary groups, although these results were not significant, given intragroup variability. However, there was a significant mean 2.37 ± 0.770 log_10_ CFU/g reduction in kidney fungal burden between the CD + FCN and KD + FCN groups (*P* = 0.0174).

To test if a KD affected blood chemistry or the population of cellular components in the invasive candidiasis model, a cardiac blood draw was conducted at the time of euthanasia to measure complete blood counts (CBCs), blood chemistry, and endpoint fluconazole concentrations. Certain white blood cell populations were lower in ketogenic diet groups (Fig. S2A). There were no consistent significant differences in blood chemistry levels across dietary groups that would indicate a significant impact on liver or kidney organ function (Fig. S2B). Interestingly, endpoint serum fluconazole levels were on average over 10 times higher in KD mice compared to CD (mean serum levels of 4.06 and 0.31 µg/mL, respectively; *P* < 0.0001).

### Fluconazole exposure is significantly increased in blood plasma and brain tissue of ketogenic diet-treated mice

**Fig 3:**
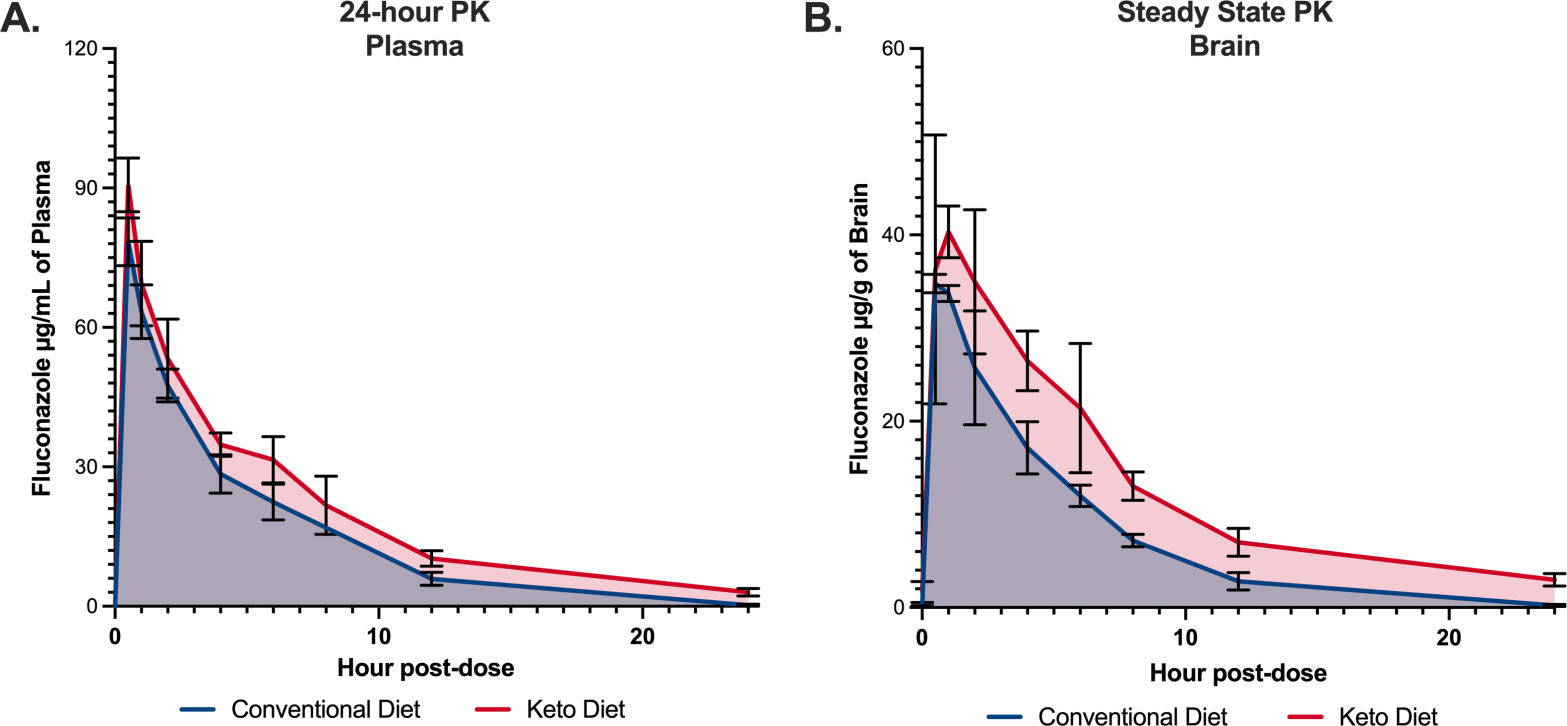
Serum and brain fluconazole levels are increased in mice given a ketogenic diet. (**A**) After a single dose of fluconazole, fluconazole serum exposure after 24 h (AUC_0–24_) for a KD was 453 µg*h/mL, versus 340 µg*h/mL in the CD group. (**B**) At steady state after 4 days of dosing, brain exposure to fluconazole (AUC_120–144_) was 310 µg*h/mL in the KD group and 185 µg*h/mL in the CD group. Fluconazole levels in both experiments were measured via liquid chromatography-mass spectrometry, and the mean of at least three mice was reported for each time point. AUC_0–24_, area under the curve from 0 to 24 h; AUC_120–144_, area under the curve from 120 to 144 h;PK, pharmacokinetic.

Given the dramatic decrease in brain fungal burden of mice on a KD and treated with fluconazole, both single-dose and steady-state pharmacokinetic (PK) studies were undertaken during *C. neoformans* infection. After dietary intervention and infection with *C. neoformans*, 80-mg/kg fluconazole i.p. was dosed either once for single-dose plasma PK analysis or after 4 days of daily fluconazole treatment for tissue steady-state analysis. Fluconazole PK for both dietary conditions was linear. After a single dose of fluconazole, drug levels were increased in KD mice at every time point, and the average total drug area under the curve from 0 to 24 h (AUC_0–24_) was increased by 33.2% on a KD at 453 µg*h/mL compared to CD at 340 µg*h/mL (Fig. 3A). The effect was even more pronounced in the steady-state model, with a 10-fold increase in brain fluconazole levels at 24 h post-dose in KD mice and a 67.6% increase in average area under the curve from 120 to 144 h (AUC_120–144_) in KD at 310 µg*h/mL versus CD conditions at 185 µg*h/mL (Fig. 3B). Cumulatively, these results indicate fluconazole exposure is significantly increased in both plasma and brain tissue of mice during ketosis.

### A ketogenic diet increases fluconazole efficacy and induces stasis at a significantly lower exposure, as quantified using dose or AUC

To better assess the relationship of fluconazole concentration and fungal burden, dose-response curves were generated for ketogenic and conventional dietary conditions. Infected KD and CD mice were treated with 40-, 80-, 160-, 240-, or 400-mg/kg fluconazole i.p. daily. Additionally, fluconazole was evaluated in KD mice at 0.05, 0.5, 5.0, 10.0, and 20.0 mg/kg i.p. daily.

**Fig 4:**
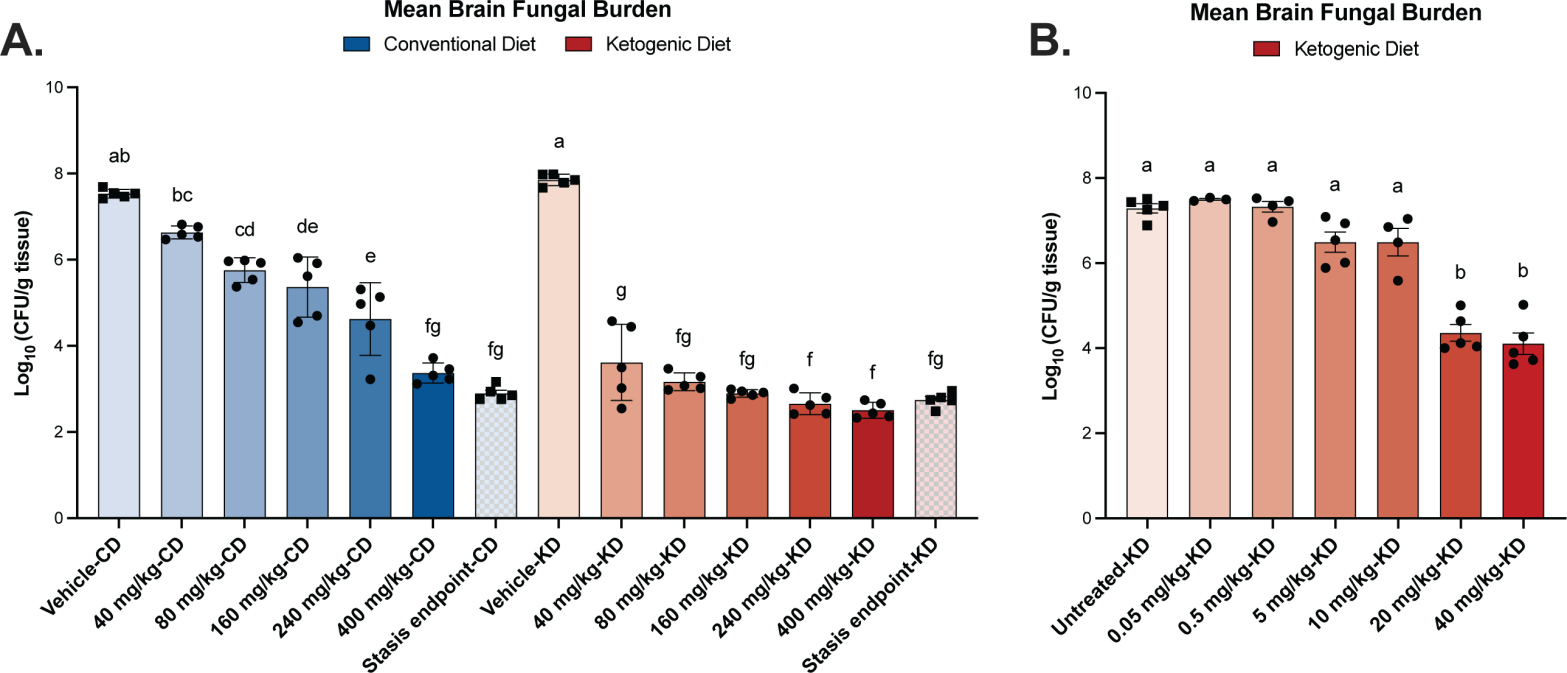
(A) Mice on a KD reach stasis at lower doses of fluconazole compared to mice on a conventional diet. Fungal burdens were equivalent for fluconazole doses of 80 and above on a KD, and all doses achieved fungal stasis. Mice on a CD displayed a stepwise reduction in yeasts as doses increased, only reaching stasis at 400 mg/kg. (**B**) The 50% efficacy level of fluconazole on a KD falls between 10 and 20 mg/kg. Mean ± SEM reported for *n* = 5 mice per group for panel A and *n* ≥ 3 mice per group for panel B. A and B panels represent independent experiments. Two-way analysis of variance with post hoc Bonferroni test.

There was no statistical difference between brain fungal burden of 80-mg/kg fluconazole on a KD (3.17 ± 0.0926 log_10_ CFU/g) and a fivefold higher dose of 400-mg/kg fluconazole on a CD (3.37 ± 0.105 log_10_ CFU/g) (Fig. 4). Additionally, there were no statistical differences in fungal burden between any KD fluconazole treatment groups over 80 mg/kg, as the mean brain fungal burdens for 80-, 160-, 240-, and 400-mg/kg fluconazole were all highly similar (3.17, 2.90, 2.66, and 2.51 log_10_ CFU/g, respectively).

A stasis endpoint—or the concentration of fluconazole necessary to prevent fungal growth after the initiation of drug treatment—was used to evaluate drug efficacy (22). One day after systemic *C. neoformans* infection, an untreated cohort from each dietary condition was euthanized, and brain fungal burden was measured to establish a stasis line (KD, 2.74 log_10_ CFU/g; CD, 2.89 log_10_ CFU/g). Estimates indicate that, on a KD, stasis is achieved at 220.41-mg/kg fluconazole (plasma area under the curve [AUC] = 1,412.8 µg*h/L), while 531.85-mg/kg fluconazole (plasma AUC = 2179.7 µg*h/L) is required to achieve stasis on a CD (Fig. 4).

**Fig 5:**
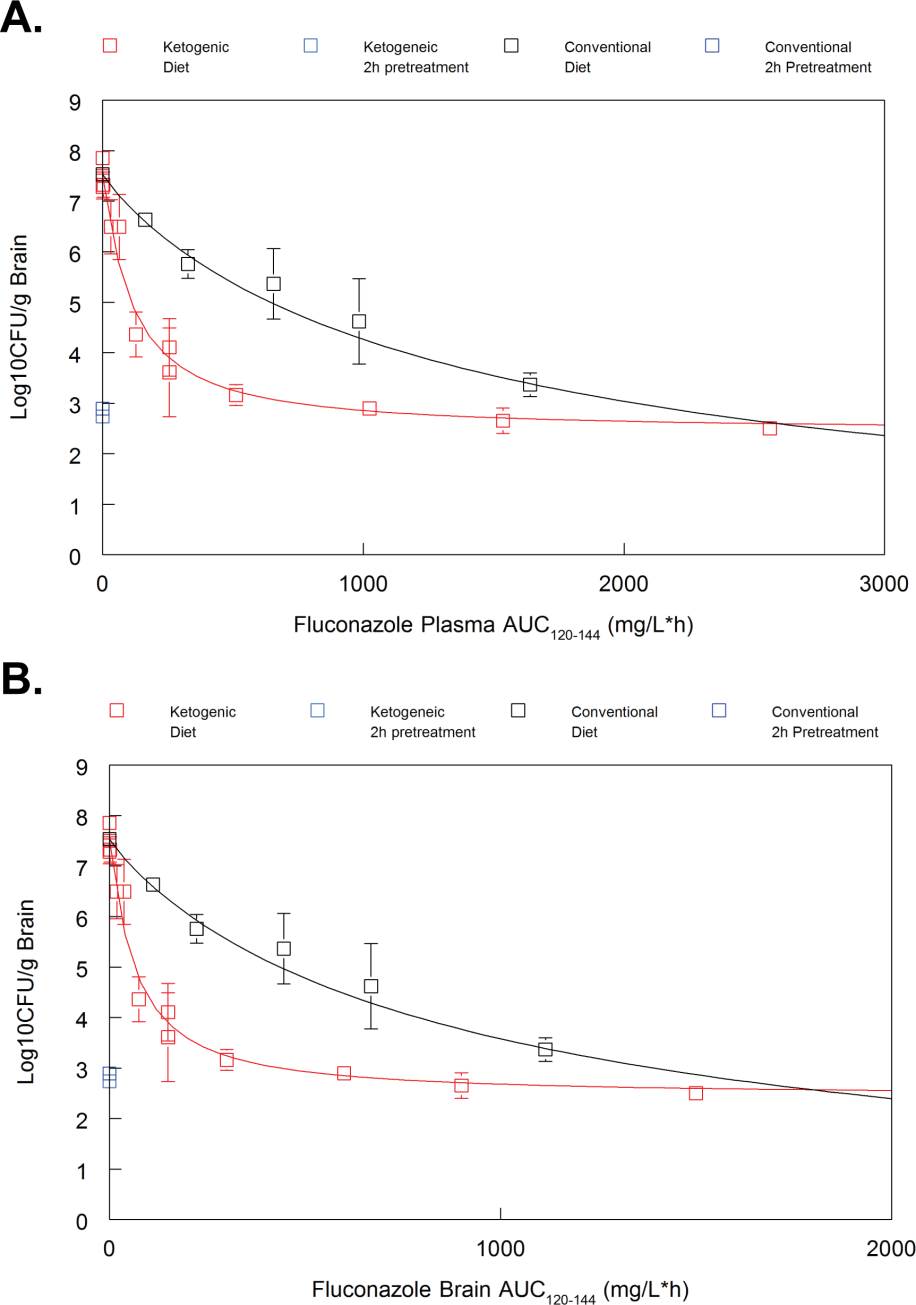
Pharmacodynamic analysis of fluconazole exposure and *C. neoformans* fungal burden. An inhibitory sigmoid Emax model was used to estimate (**A**) plasma AUC versus effect relationship and (**B**) brain AUC versus effect relationship.

A pharmacodynamic analysis determined that neither plasma nor brain PK changes were exclusively responsible for the observed fungal burden differences across dietary groups (Fig. 5A and B). When fungal burden was assessed in relation to fluconazole AUC, there was still a significant gap between dietary groups. This indicates that an unknown pharmacodynamic effect, in addition to fluconazole exposure changes, is responsible for the dramatic decrease in fungal burden observed on a KD compared to a CD. A summary of parameters can be found in Table 1. Furthermore, the consistency across plasma and brain analyses indicates these differences are not driven by changes in central nervous system (CNS) partitioning.

**TABLE 1:** Mean values for each parameter from the pharmacokinetic/pharmacodynamic linked model

Parameters^*a*^ (units)	Conventional diet cohort mean	Ketogenic diet cohort mean
Ka (h^−1^)	3.168	10.66
SCL (L/h)	0.0061	0.0039
Vc (L)	0.0009	0.0104
Kcp (h^−1^)	1.831	7.924
Kpc (h^−1^)	0.2896	9.925
Kcb (h^−1^)	0.028	1.77
Kbc (h^−1^)	9.258	2.867
Vbrain (L)	0.0000042	0.011

^*a*^ Ka, absorption rate; Kcp, Kpc, Kcb, and Kbc, first-order intercompartmental rate constants connecting the central, peripheral, and brain compartments; SCL, first-order fluconazole clearance from the central compartment; Vbrain, brain volume; Vc, central compartment volume.

### The interaction between a ketogenic diet and fluconazole is rapidly induced

**Fig 6:**
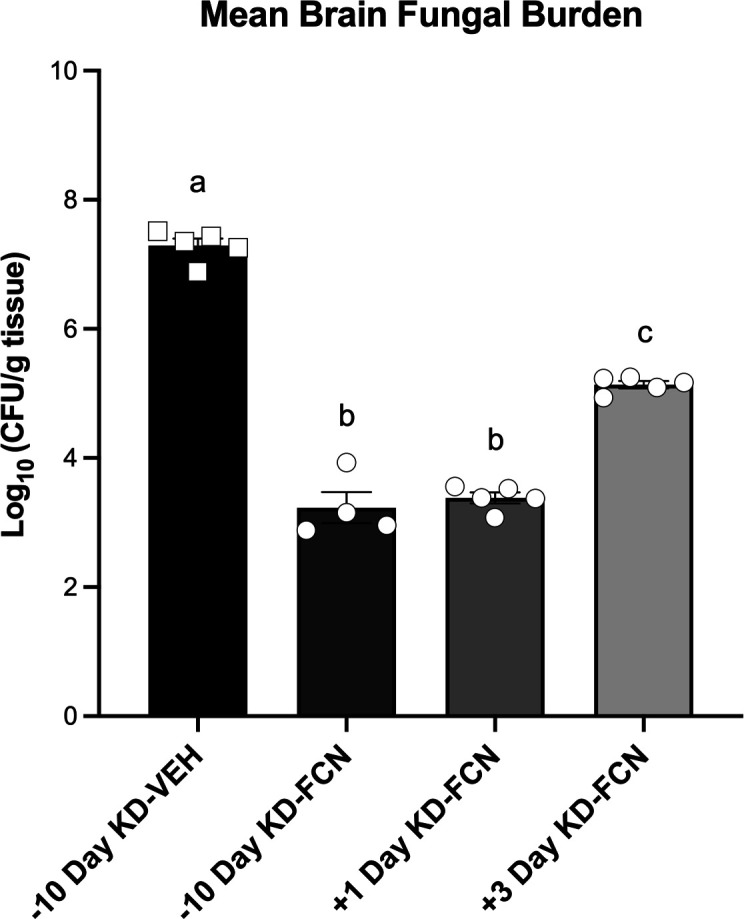
A delayed switch to a KD, 24 h post-inoculation, is just as effective as 10 days of dietary induction. Brain fungal burden in mice begun on a KD 24 h post-inoculation and treated with fluconazole (FCN) was statistically equivalent to those receiving a KD 10-day pre-inoculation. Mice switched to a ketogenic diet on day 3 after infection showed increased fungal burden compared to mice that started a ketogenic diet 24 h post-infection but had lower fungal burden than mice on a KD without fluconazole (*P* < 0.0001 for both). Mean ± SEM reported for ≥4 mice per group from one independent experiment. One-way analysis of variance with post hoc Tukey test was used.

We next wanted to evaluate how quickly a KD can affect brain fungal burden during *Cryptococcus* infection. Mice were started on a KD 10 days before infection (15 days total on a KD), 24 h post-inoculation (4 days total on a KD), and 72 h post-inoculation (2 days total on a KD) and were treated with 80-mg/kg fluconazole. Brain fungal burden was assessed 5 days post-inoculation. The mean brain fungal burden of mice started on a KD 10 days before infection and 24 and 72 h post-inoculation were 3.23, 3.38, and 5.14 log_10_ CFU/g, respectively (Fig. 6). There was no difference in mean brain fungal burden of mice on a KD 10 days before infection and mice on a KD 24 h post-inoculation. However, a transition to a KD at 72 h post-inoculation failed to dramatically reduce the brain fungal burden when compared to the 10-day pre-inoculation or 24 h post-inoculation dietary groups (*P* < 0.0001 for both). We then asked how long the effect of the KD lasted. We administered a KD to mice for 10 days and subsequently switched them from a KD to a CD at 8 h post-inoculation and treated them with fluconazole. These mice had brain fungal burden levels equivalent to those on a CD for the duration of the study, indicating a lack of a protective effect of a KD or that the effect is rapidly reversed once mice are fed a CD (Fig. S3).

Given the rapid onset of this effect, we tested whether the diet itself may possess some antifungal properties. Crude extracts of the diet were tested in disc diffusion assays. However, the diet extracts did not appear to have any antifungal activity, which aligned with our observation that the diet alone did not have an effect on fungal burden (data not shown).

### Butyrate synergizes with fluconazole *in vitro*, but activity failed to be replicated *in vivo*

Given that several studies have identified specific ketone bodies as the mechanism underpinning amelioration of certain disease states on a KD, we evaluated whether β-hydroxybutyrate (BHB) and/or butyric acid possessed inherent antifungal activity or demonstrated an interaction with fluconazole (supplemental materials) (23–25). Butyric acid, as the salt sodium butyrate (SB), showed modest antifungal activity, with a minimum inhibitory concentration (MIC_50_) of 25 mM, while BHB demonstrated little-to-no activity (MIC_50_ = 360 mM). SB appeared to synergize with fluconazole, with combinatorial MICs of 0.39 mM and 0.25 µg/mL, respectively, and a fractional inhibitory concentration index (FICI) of 0.2656. To see if supplementation with SB could replicate the effects of the KD, and given that we saw the effect of the KD when beginning the diet 24 h post-infection, we administered SB by two different means, intraperitoneally and orally, starting at 24 h post-infection. However, the *in vitro* effects could not be replicated by directly administering SB, either intraperitoneally or orally via drinking water (Fig. S4A and B). There was no significant difference in fungal burden between untreated mice and those treated with SB via either route, and the combination of SB and fluconazole did not show enhanced activity *in vivo* compared to fluconazole alone. Although we cannot rule out the possibility of other ketone bodies driving the reduction of fungal burden *in vivo*, it appears that butyric acid cannot drive this effect in isolation at the doses we used.

### Endpoint immune responses are largely similar between ketogenic and conventionally fed mice

Previous reports have found that a ketogenic diet is associated with an altered immune response (7, 26–28). We analyzed serum cytokine expression in mice during *Cryptococcus* infection on a CD or KD diet at the study endpoint, 6 days post-inoculation. Blood was collected via cardiac puncture and measured for levels of interleukin-2 (IL-2), IL-4, IL-5, IL-6, IL-9, IL-10, IL-13, IL-17A, IL-18, IL-22, IL-23, IL-27, IL-22 p70, interferon gamma (IFN-γ), tumor necrosis factor alpha (TNF-α), and granulocyte-macrophage colony-stimulating factor (GM-CSF). Differences in cytokine levels between KD and CD dietary groups were of particular interest to determine if immune response changes may be driving the significant decrease observed in fungal burden in KD +FCN groups.

**Fig 7:**
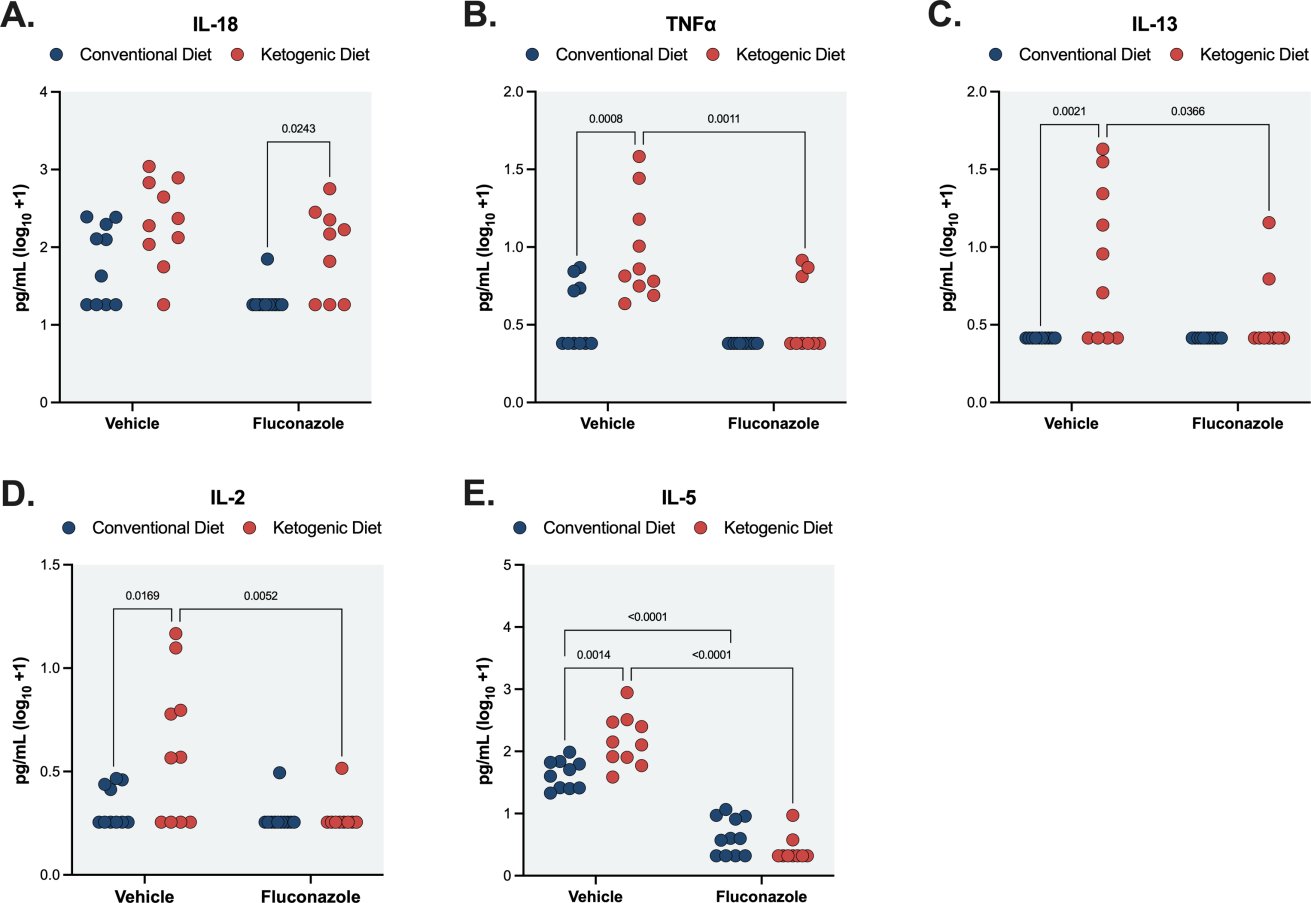
Serum cytokine profiles of mice infected with *C. neoformans*, collected 6 days post-infection display several slight differences between dietary cohorts. (**A**) Mice on a KD showed an increased expression of IL-18 compared to a CD during fluconazole treatment (*P* = 0. 0.0243). (**B**) TNF-α expression was increased in vehicle-treated KD mice compared to CD (*P* = 0.0008). (**C**) IL-13 expression was enhanced in vehicle-treated KD mice compared to CD (*P* = 0.0021). (**D**) IL-2 expression was increased in vehicle-treated KD mice compared to CD (*P* = 0.0169). (**E**) IL-5 expression was significantly increased in vehicle-treated KD versus CD mice (*P* = 0.0014). Fluconazole treatment decreased IL-5 expression overall within both dietary groups (CD <0.0001, KD <0.0001). Cytokine concentrations were measured for at least nine mice per group and (log_10_ +1) transformed. Two-way analysis of variance with post hoc Tukey test was used.

Several cytokines showed no significant trends (IL-22, IL-27, and IL-17A) or were under the limit of detection (IL-9, IL-10, IL-23, IL-22 p70, and GM-CSF). However, IL-18 expression was increased in the KD + FCN cohort compared to CD + FCN (*P* = 0.0243), although many CD + FCN samples were at or below the lower limit of quantification (LLOQ) (Fig. 7A). Despite no difference in tissue fungal burden, TNF-α, IL-13, IL-2, and IL-5 had significantly increased expression in the KD + VEH groups compared to the CD + VEH group, and their expression was decreased in the KD cohort upon fluconazole treatment (Fig. 7B through E). IFN-γ and IL-6 expression were decreased in fluconazole-treated groups compared to the vehicle controls within each dietary condition, while IL-4 expression was decreased in the KD + FCN group compared to the KD + VEH control, with some samples falling below the lower LLOQ (Fig. S5). Given the significant differences in fungal burden across dietary and treatment groups, some changes in cytokine expression may be a result of stronger anticryptococcal immune responses in groups with high fungal burden. In summary, differences in cytokine levels under different dietary treatment conditions were observed for IL-18, TNF-α, IL-13, IL-2, and IL-5, while IFN-γ, IL-6, and IL-4 displayed differences within dietary groups.

## DISCUSSION

*Cryptococcus neoformans* and *Candida albicans* are two of the most common causative species of opportunistic mycoses and have mortality rates that can exceed 60%, depending on healthcare settings and specific patient populations (3, 29). Currently, treatment for cryptococcal meningitis involves three stages—induction, consolidation, and maintenance therapies—involving three classes of antifungals and lasting up to 1 year. The complex regimen and associated toxicities led the WHO to adopt a streamlined treatment plan for resource-limited healthcare settings in 2022 (30, 31). Invasive candidiasis is primarily treated with echinocandins, but this class of antifungals achieves low CNS partitioning (32). As a result of the increase in drug resistance rates and reduced fungicidal activity, triazole treatments for invasive candidiasis are recommended only in less severe cases or where azole susceptibility is likely. However, prescribing patterns for IFIs differ between high income (HIC) and low- and middle-income countries (LMIC). LMICs were found to rely on fluconazole more than HICs (33). This is likely a direct result of inaccessibility and costliness of alternate treatments. Fluconazole is available worldwide (in 2016, 27% of countries did not have access to amphotericin B, and flucytosine was inaccessible in 78% of countries) and is relatively inexpensive compared to other antifungals, making it a first-and-only line treatment for IFIs in areas where no alternatives exist (34). While there are currently several antifungal drugs in clinical development, there is an acute need for cost-effective, accessible, and efficacious antifungal therapeutics. Here we present compelling evidence for fluconazole treatment potentiation using a ketogenic diet.

We evaluated whether a dietary intervention, with and without a standard regimen of fluconazole, could significantly decrease fungal burden of two major yeast pathogens compared to a conventional diet. A KD, which induces ketosis by restricting carbohydrate intake to 5% of daily calories, was selected due to the hypothesized effect on fungal glucose metabolism. In murine models of systemic cryptococcosis, a KD alone showed no difference in brain or lung fungal burden compared to a CD. However, KD, in combination with fluconazole, demonstrated a significant 2.66 ± 0.289 log_10_ CFU/g decrease in brain cryptococcal burden compared to fluconazole-treated CD mice. The lung showed a less dramatic, albeit still significant, decrease in fungal burden between fluconazole-treated KD and CD groups of 1.72 ± 0.399 log_10_ CFU/g. These results indicate that, while a KD alone may not affect *C. neoformans* fungal burden, it significantly potentiates the effect of fluconazole during infection.

To determine whether the potentiation of fluconazole by a KD was specific to *C. neoformans*, the treatment model was evaluated in a systemic *C. albicans* infection. Similarly, while a KD alone had no measurable effect, it resulted in a 2.37 ± 0.770 log_10_ CFU/g decrease in mean kidney fungal burden when combined with fluconazole compared to a CD. Therefore, KD-induced fluconazole potentiation occurs across different sites of infection and against multiple fungal pathogens. Further work is required to determine if this treatment combination would be efficacious in superficial sites of infection against other fungal pathogens, including endemic mycoses and dematiaceous molds, in chronic-infection models at prolonging overall survival and in combination with other standard therapies like amphotericin B and caspofungin.

Fluconazole pharmacokinetics were altered on a KD, with a dramatic enhancement of peak plasma and brain tissue levels, as well as an overall increase in drug exposure after a single dose and through steady-state analyses. Furthermore, maximum fluconazole efficacy, measured by a stasis endpoint, was achieved at significantly lower drug concentrations on a KD compared to a CD (220.41-mg/kg fluconazole versus 531.85-mg/kg fluconazole, respectively). A stasis endpoint, or the drug concentration that fully inhibits fungal growth, has been identified as a valuable pharmacological target with physiological relevance (22). Lower doses of fluconazole under ketosis clearly provide rapid control of yeast growth *in vivo*.

Fluconazole monotherapy is no longer recommended due to the emergence of resistance, reduced fungicidal activity, and poor clinical outcomes (30, 35). Often, fluconazole cannot be dosed in high-enough concentrations to provide sufficient time above MIC values for effective antifungal activity, leaving hetero-resistant or tolerant subpopulations of yeast to re-establish persistent infection (35). While efforts have been made to increase therapeutic concentrations of fluconazole (doses up to 2,000 mg/day), the tolerability and PK of these doses are not well understood (36). The mechanism driving increased serum and tissue fluconazole concentrations on a KD remains unknown, and a KD does not appear to change serum concentrations of common antiepileptic drugs in the long term (37). However, bioavailability of oral administration of other triazoles can be both positively (posaconazole) or negatively (voriconazole) affected by co-administration with fatty foods (38, 39). Modulating drug exposure in a patient, possibly through dietary intervention, may present a novel way to increase time above MIC, thereby boosting fluconazole efficacy and decreasing development of resistant subpopulations within a host.

Pharmacodynamic analysis of both plasma and brain fluconazole exposure indicate that PK changes are not solely responsible for the increase in fluconazole efficacy observed. One explanation may involve differing growth rates of yeast cells across dietary groups and subsequent effects on fluconazole activity. Cellular growth rate is known to influence antifungal efficacy *in vivo*, and *in vitro* work has found that slower-growing cells are often more resistant to antimicrobial drugs (40, 41). In *C. neoformans*, changes in nutrient availability have been shown to affect growth rate, and expression of fatty acid degradation proteins is decreased in viable but non-culturable, or latent, cells (42). A KD may be altering the nutrient composition of host tissues, thereby affecting transcription of metabolic enzymes and fungal growth rate. However, further work is necessary for determining the underlying mechanism of this significant pharmacodynamic effect.

We have also determined that a KD enhances fluconazole activity when introduced 24 h post-inoculation, totaling just 4 days on a KD. A KD-induced potentiation of fluconazole, therefore, may be independent of the host metabolic switch to ketosis. Previous studies have determined that the effect of a KD may be distilled into a single metabolite, such as β-hydroxybutyrate (23–25). Although we found that butyric acid can potentiate fluconazole antiyeast activity *in vitro*, we could not replicate these findings *in vivo* with exogenous butyric acid. Additionally, while the 3-day delay in dietary induction showed increased fungal burden compared to a 1-day delay, this group was on a KD totaling just 2 days before CFU quantification. A longer duration study may be necessary to determine efficacy during chronic infection. However, the rapid antifungal effect of KD after only several days has implications for a clinical setting, in which a dietary intervention can be introduced during an established infection. For instance, several studies have found that a KD is a feasible treatment intervention for pediatric epilepsy in LMICs, and success is increased when administered in hospital settings (43–46). Furthermore, a KD was found to significantly alter the human immune system, microbiome, and metabolome after just 2 weeks of therapy (47). Therefore, we believe further investigation of a KD is warranted to determine its applicability for improved care of fungal infections in resource-limited healthcare settings.

Previous studies investigating the interaction between KD and infection found the beneficial effect of a KD was largely driven by a repression of pathogenic inflammatory immune responses and an enhancement of immunoprotective cell populations (7, 26, 27, 48). Specifically, KD-induced proliferation of γδ T cells enhances antiviral immunity and bolsters tissue barrier integrity against influenza and coronaviruses (7, 27). Interestingly, at day 6 post-inoculation, most cytokines studied were not drastically or consistently different across KD and CD groups, especially the fluconazole-treated groups that showed the most dramatic differences in mean fungal burden. IL-18 was the only measured cytokine whose expression differed between fluconazole-treated dietary groups, with an increase in KD-fluconazole mice. IL-18 enhances the Th1 antifungal immune response through IFN-γ stimulation and induces IL-17 production by γδ T cells (49, 50). Previous studies have found that IL-18 deficiency results in increased mortality and fungal burden during systemic *C. neoformans* and *C. albicans* infection, and supplementation of IL-18 can improve outcomes in models of disseminated candidiasis (51–54). Additional work will be needed to identify any differences in cytokine expression in target tissues like the brain and to quantify possible differences in immune cell subpopulations, including γδ T cells, that correlate with improved host control of yeast growth during treatment.

In summary, this work represents, to our knowledge, the first time that a KD has been shown to rapidly increase the efficacy of antifungal therapy for invasive fungal infections. A ketogenic dietary intervention enhances fluconazole activity during murine models of cryptococcal meningitis and disseminated candidiasis. A KD had a significant impact on fluconazole drug exposure and pharmacodynamics in the host and was found to maximize antifungal efficacy at significantly lower fluconazole concentrations. Future work will be necessary to isolate the compounds or processes responsible for this therapeutic effect and to understand the potential extent of activity regarding poorly tolerated antifungals and against other deadly mycoses. However, this strategy presents a promising avenue for improving antifungal therapeutic strategies against cryptococcal and *Candida* disease where only fluconazole is available.

## MATERIALS AND METHODS

### Strains used in this study

The *Cryptococcus neoformans* strain H99 and *Candida albicans* strain SC5314 were used in these studies. All strains were streaked from a –80°C glycerol stock onto yeast extract-peptone-dextrose (YPD) media prior to each experiment. Individual colonies were selected and cultured in YPD broth overnight at 30°C. The yeast cells were harvested, washed three times in phosphate-buffered saline (PBS), pH 7.4, and then counted using a Cellometer T4 Cell Counter. The inoculum cell concentration was adjusted to the target concentration by dilution in PBS.

### Animal use and care

Male CD1 mice (Charles River Laboratories, Wilmington, MA) weighing approximately 19–21 g were used for the animal studies. Food and water were provided *ad libitum*.

### Delayed treatment model of *C. neoformans* infection

The *Cryptococcus* delayed treatment model was used as described, with two modifications that included a diet change 10 days prior to inoculation and an endpoint of 6 days post-inoculation (55). Ten days prior to infection and continuing through euthanasia, the dietary intervention group (*n* = 20) was fed a ketogenic diet (Bioserve AIN-76A-modified, high-fat paste), while the control groups were fed a conventional chow diet (Lab Diet 5008) (*n* = 20). Blood-glucose and blood-ketone measurements were taken 1 day before dietary intervention (day −10), 1 day before infection (day −1), and 1 day before euthanasia (day 5) via tail-vein stick with a FreeStyle Precision xtra monitoring system and compatible glucose and ketone test strips (Abbott Laboratories) (56).

On the day of infection, an inoculum of ~5 × 10^4^ cryptococcal yeast cells was prepared in 100 µL of PBS, and mice were inoculated via lateral tail-vein injection. Approximately 24 h post-inoculation, half of each dietary group (*n* = 10) was treated with 80-mg/kg fluconazole i.p. (Sagent Pharmaceuticals) once daily. The remaining untreated mice were dosed with sterile PBS i.p. once daily. All groups were euthanized 6 days post-inoculation via CO_2_ inhalation. The brain and left lung were processed for fungal burden analysis as previously described (55). CFU per gram of tissue was calculated and log_10_ transformed.

### Dose-exposure-response relationships

Mouse handling and inoculations were performed as described above. To establish the stasis point, or baseline fungal burden without therapy, brain fungal burden was measured for five mice per dietary group at 24 h post-inoculation. To evaluate the fluconazole dose-fungal burden response relationship, five mice per dietary group were treated with 40-, 80-, 160-, 240-, and 400-mg/kg fluconazole i.p., beginning 24 h post-inoculation and concluding 5 days post-inoculation. A stock solution of 10-mg/mL fluconazole (Thermo Scientific) in sterile PBS and 4% dimethyl sulfoxide (DMSO) was sterile filtered and sonicated with gentle heat (42°C) before use. Untreated mice were administered the 4% DMSO + PBS vehicle.

To estimate the dose-exposure-response of fluconazole on a ketogenic diet, the experiment was replicated with fluconazole concentrations of 0.05, 0.5, 5.0, 10.0, 20.0, and 40.0 mg/kg using a stock solution of 2-mg/mL fluconazole in sodium chloride (Sagent Pharmaceuticals). Brain fungal burden was assessed at day 5 post-inoculation due to progression of infection in untreated controls.

### Delayed dietary inductions

Mice were fed either a ketogenic diet or conventional diet beginning 10 days prior to infection. Inoculations were performed, and the conventionally fed mice began a ketogenic diet at 24 or 48 h post-inoculation (*n* = 5). Each group was dosed with 80-mg/kg fluconazole i.p., beginning 24 h post-inoculation. Mice that were pre-treated with a KD 10 days before infection were treated with either a PBS vehicle control or 80-mg/kg fluconazole. For all groups, fungal burden was assessed 5 days post-inoculation.

### Delayed treatment model of *C. albicans* infection

For *Candida* experiments, mice were inoculated with ~2 × 10^5^ yeast cells in 100 µL via lateral tail-vein injection, as described previously (57). Half of each dietary cohort (*n* = 10) was treated with 0.25-mg/kg fluconazole i.p. daily. Blood glucose and ketone measurements were taken 1 day prior to diet initiation, inoculation, and euthanasia. Kidney fungal burden was measured at 8 days post-inoculation. Blood was collected via cardiac puncture for CBC and blood chemistry analysis, and blood serum was isolated for endpoint fluconazole measurement.

### Pharmacokinetic/pharmacodynamic analysis

#### Single-dose experiment

Ketogenic and conventional diet-fed mice (three to five per time point) were dosed once with 80-mg/kg fluconazole i.p. 24 h post-inoculation. Approximately 0.5- to 0.8-mL blood was collected via cardiac puncture at 0.5, 1.0, 2.0, 4.0, 8.0, 12.0, and 24.0 h post-dose. Blood was transferred into a K_2_EDTA tube (BD) and spun at 5,000 × *g* for 15 minutes at 4°C. Plasma was collected and stored in a 2-mL screw-cap tube and frozen at –80°C until analyzed for fluconazole levels. Fluconazole concentrations were measured via liquid chromatography-mass spectrometry by the Duke Proteomics and Metabolomics Core.

#### Steady-state experiment

On day 5 post-inoculation, ketogenic and conventional diet-fed mice (three to five per time point) were dosed with 80-mg/kg fluconazole i.p. Mice were then euthanized at 0, 0.5, 1.0, 2.0, 4.0, 8.0, 12.0, and 24.0 h post-dose and brain extracted. Whole brain was transferred to a 7-mL bead blaster CK-14 Precellys homogenization tubes (Bertin Corp), frozen on dry ice, and stored at –80°C for subsequent tissue analysis. Fluconazole concentration was measured via ultra-performance liquid chromatography-tandem mass spectrometry on a Waters TQ-S mass spectrometer by the Duke Proteomics and Metabolomics Core and data analysis using Skyline (supplemental material).

### Pharmacokinetic-pharmacodynamic modeling

A four-compartment PK model was fitted to the pharmacokinetic data, which took the form


$$\begin{array}{c} XP(1)=-Ka^{*}X(1)\\ XP(2)=Ka^{*}X(1)-(SCL/Vc)+Kcp{)}^{*}X(2)+Kbc^{*}X(4)+Kpc^{*}X(3)-Kcb^{*}X(2)\\ XP(3)=Kcp^{*}X(2)-Kpc^{*}X(3)\\ XP(4)=Kcb^{*}X(2)-Kcb^{*}X(4) \end{array}$$

where Ka is the first-order absorption constant (h^−1^); SCL (L/h) is the first-order clearance of fluconazole from the central compartment; Vc (L) is the volume of the central compartment; and Kcp, Kpc, Kcb, and Kbc (h^−1^) are the respective first-order intercompartmental rate constants connecting the plasma to the peripheral compartment and brain. *X*(1), *X*(2), *X*(3), and *X*(4) represent the mass of fluconazole (mg) in the gut, central compartment, peripheral compartment, and brain, respectively. Equations 1, 2, 3, and 4 represent the rate of change of fluconazole (mass) in the respective compartments.

There were two output equations to estimate fluconazole concentrations in the plasma and brain, given by *Y*(1) and *Y*(2), respectively:


$$\begin{array}{c} Y(1)=X(2)/Vc\\ Y(2)=X(4)/Vbrain \end{array}$$

where Vbrain is the volume of the brain.

The PK model was implemented in ADAPT5, and the model was fitted to the data using the maximum likelihood estimator. The mean of the observations of *n* = xx mice per dose-timepoint in plasma and brain was used. The area under the concentration time curve was estimated using integration.

#### Pharmacodynamics

The final solution of the PK model (above) was used to estimate the AUC for each regimen in mice receiving a conventional versus ketogenic diet. The AUC_120–144_ was used. Dose versus effect and AUC versus effect relationships were described using an inhibitory sigmoid Emax model, which took the form


$$Effect\;=\;Econ-(Emax^{*}(exposure{)}^{H}/({E_{50}}^{H}+(exposure{)}^{H}))$$

The model was implemented in ADAPT version 5 and fitted to the data using weighted least squares. The data were weighted by the inverse of the observed variance.

### SB treatment during *C. neoformans* infection *in vitro* and *in vivo*

Mouse inoculations were performed as described above. At 24 h post-inoculation, SB dosing began either (i) intraperitonially at 0.8 or 1.2 g/kg, with and without 80-mg/kg fluconazole i.p., or (ii) via drinking water provided *ad libitum* at 100 mM*,* with and without 80-mg/kg fluconazole i.p. Fungal burden was assessed on day 6 post-inoculation for all groups.

### Cytokine analysis

On day 7 post-inoculation, approximately 0.5–1.0 mL of blood was collected via cardiac puncture immediately following euthanasia. Blood was transferred into a 1.7-mL microfuge tube and spun at 10,000 × *g* for 15 minutes at 4°C. Approximately 65 µL of serum was collected and stored at –80°C. Neat samples were analyzed in duplicate, excluding four samples that were run as singlets due to low volume, at first thaw with the Th1/Th2/Th9/Th17/Th22/Treg Cytokine 17-Plex Mouse ProcartaPlex Panel (Invitrogen) by the Immunology Unit of the Duke Regional Biocontainment Laboratory. A 7-point curve was generated for each cytokine, and values falling below the LLOQ were assigned one-half of the LLOQ. No samples exceeded the upper limits of quantification.

### Statistical analyses

Fungal burden (CFU/g tissue) was log transformed and then analyzed using a two-way analysis of variance (ANOVA) with post hoc Bonferroni test. The multiplicity-adjusted *P* value was reported and mean ± SEM graphed. Blood glucose and ketones were analyzed using a mixed effects model (non-parametric repeated measures two-way ANOVA) with Geisser-Greenhouse correction and post hoc Tukey test. Mean ± SD was graphed. Endpoint fluconazole levels during *C. albicans* infection were compared using a Mann-Whitney test. CBC and blood chemistry were analyzed using a two-way ANOVA with post hoc Tukey test. The multiplicity-adjusted *P* value was reported, and mean ± SD values were graphed. Cytokine concentrations were (log_10_ +1) transformed due to skewness and subsequently analyzed with a two-way ANOVA and post hoc Tukey test. GraphPad Prism version 10.1.1 for Mac OS X (GraphPad Software, Boston, MA) was used for the statistical analyses listed above.

## SUPPLEMENTAL MATERIAL

Figure S1 mbio.00649-24-s0001.tiff Mice have lowered glucose levels and reach a state of ketosis prior to and throughout infection with *C. neoformans* and *C. albicans* on a KD.

Figure S2 mbio.00649-24-s0002.tiff There are several differences in complete blood cell counts and blood chemistry between KD and CD-fed mice.

Figure S3 mbio.00649-24-s0003.tiff The effect of a KD in combination with fluconazole is rapidly lost upon reversion to a CD.

Figure S4 mbio.00649-24-s0004.tiff While sodium butyrate synergized with fluconazole *in vitro,* its antifungal effects were unable to be replicated *in vivo*.

Figure S5 mbio.00649-24-s0005.tiff Serum cytokine profiles of mice infected with *C. neoformans*, collected 6 days post-infection, display some differences between vehicle and fluconazole-treated groups within each dietary cohort.

Supplemental methods and legends mbio.00649-24-s0006.docx Additional experimental details and figure legends.

ASM does not own the copyrights to Supplemental Material that may be linked to, or accessed through, an article. The authors have granted ASM a non-exclusive, world-wide license to publish the Supplemental Material files. Please contact the corresponding author directly for reuse.
