# Supplementary figures and images for: A ketogenic diet enhances fluconazole efficacy in murine models of systemic fungal infection
Source: mBio. 2024 Apr 15;15(5):e00649-24. doi: 10.1128/mbio.00649-24 (PMC11077957; doi:10.1128/mbio.00649-24)

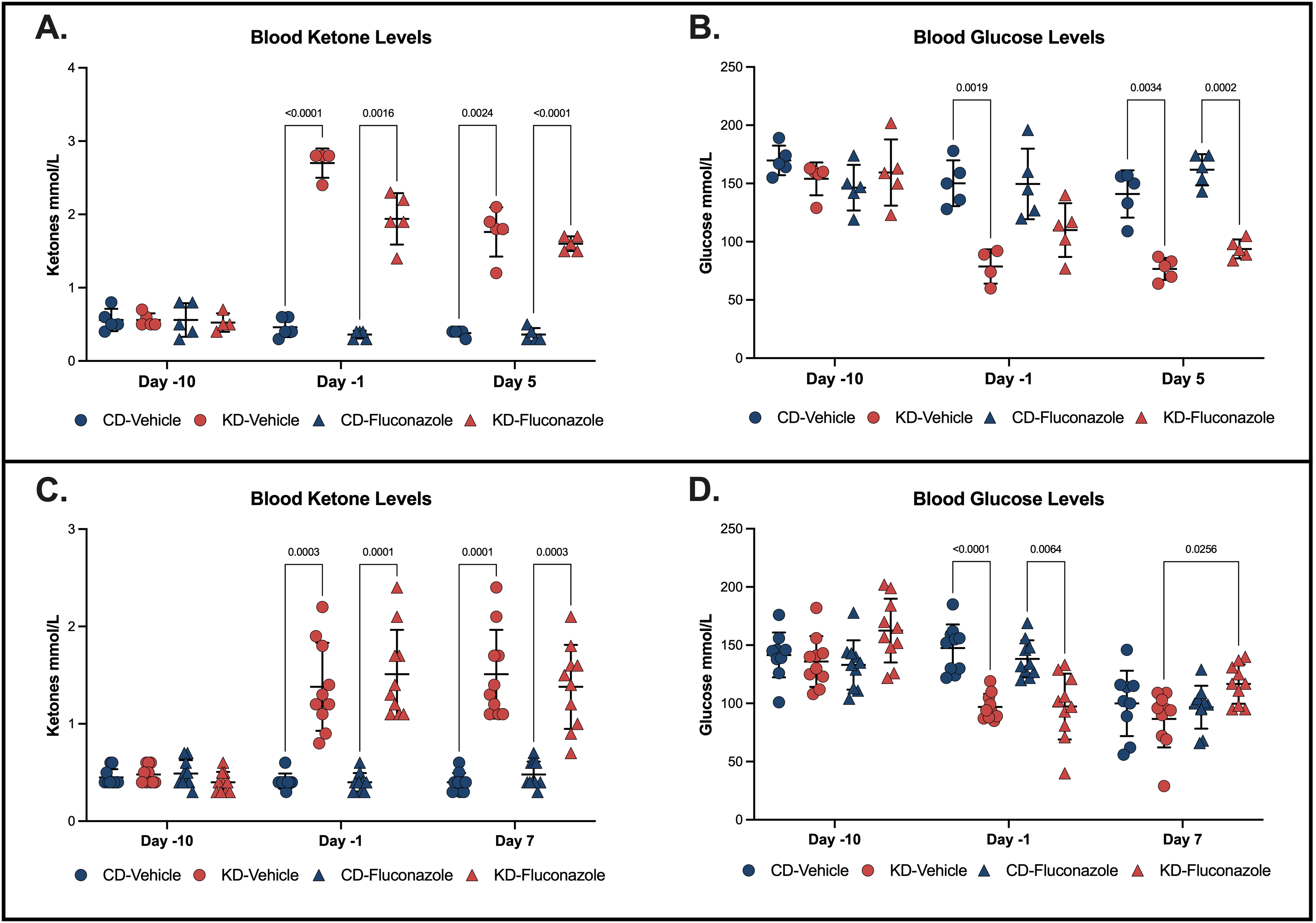

Supplement: Figure S1 — Mice have lowered glucose levels and reach a state of ketosis prior to and throughout infection with C. neoformans and C. albicans on a KD. [file mbio.00649-24-s0001.tiff]

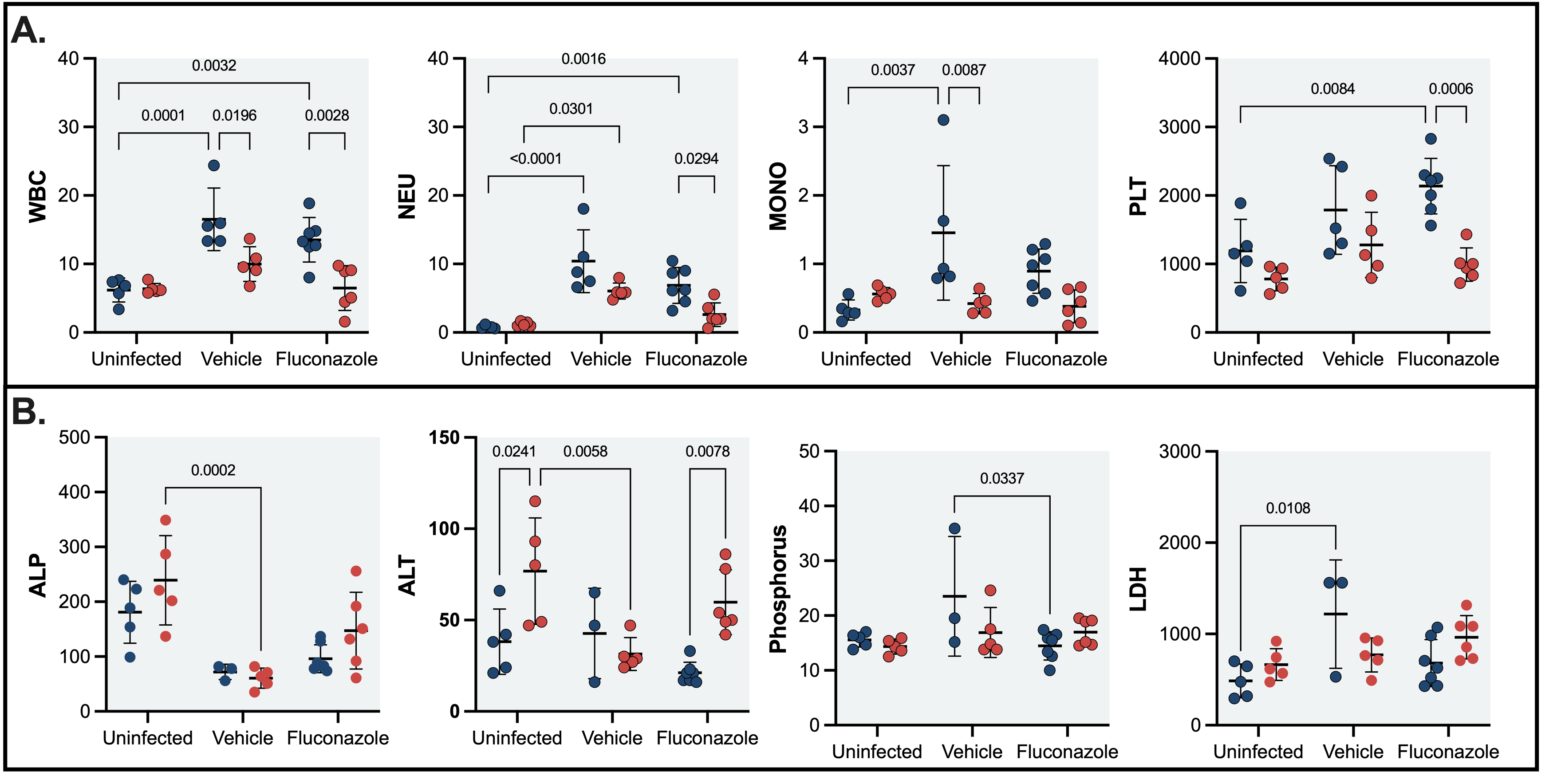

Supplement: Figure S2 — There are several differences in complete blood cell counts and blood chemistry between KD and CD-fed mice. [file mbio.00649-24-s0002.tiff]

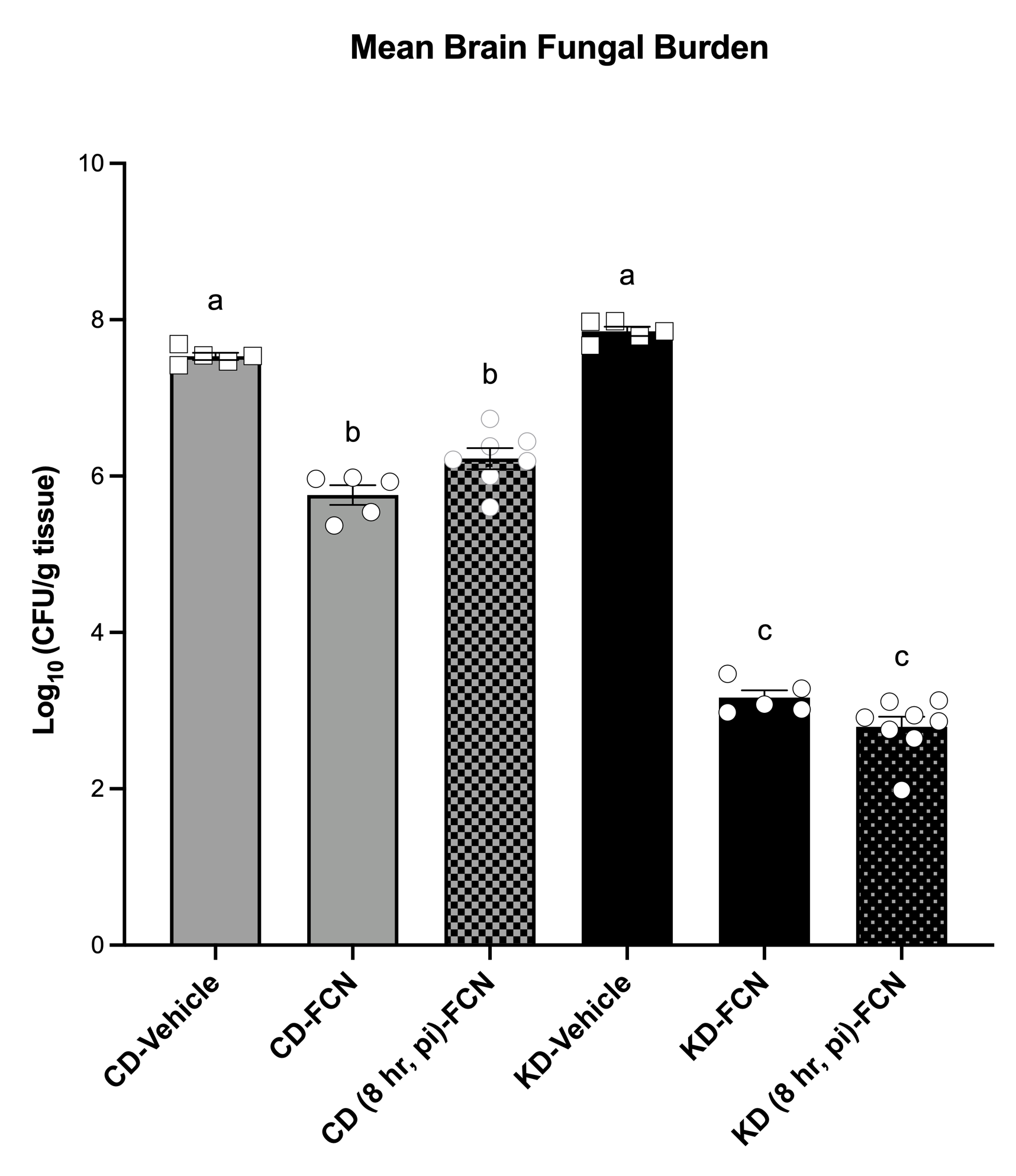

Supplement: Figure S3 — The effect of a KD in combination with fluconazole is rapidly lost upon reversion to a CD. [file mbio.00649-24-s0003.tiff]

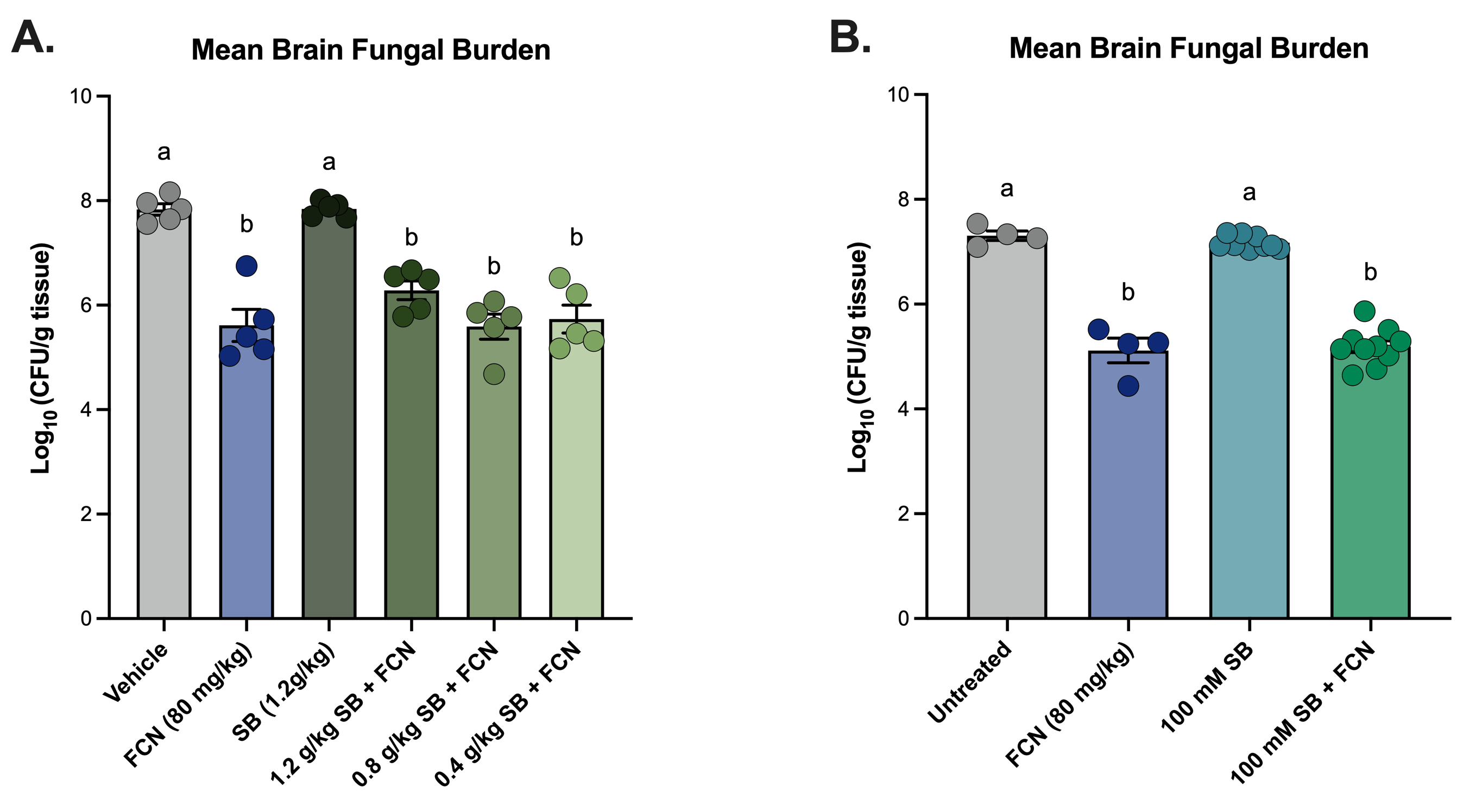

Supplement: Figure S4 — While sodium butyrate synergized with fluconazole in vitro, its antifungal effects were unable to be replicated in vivo. [file mbio.00649-24-s0004.tiff]

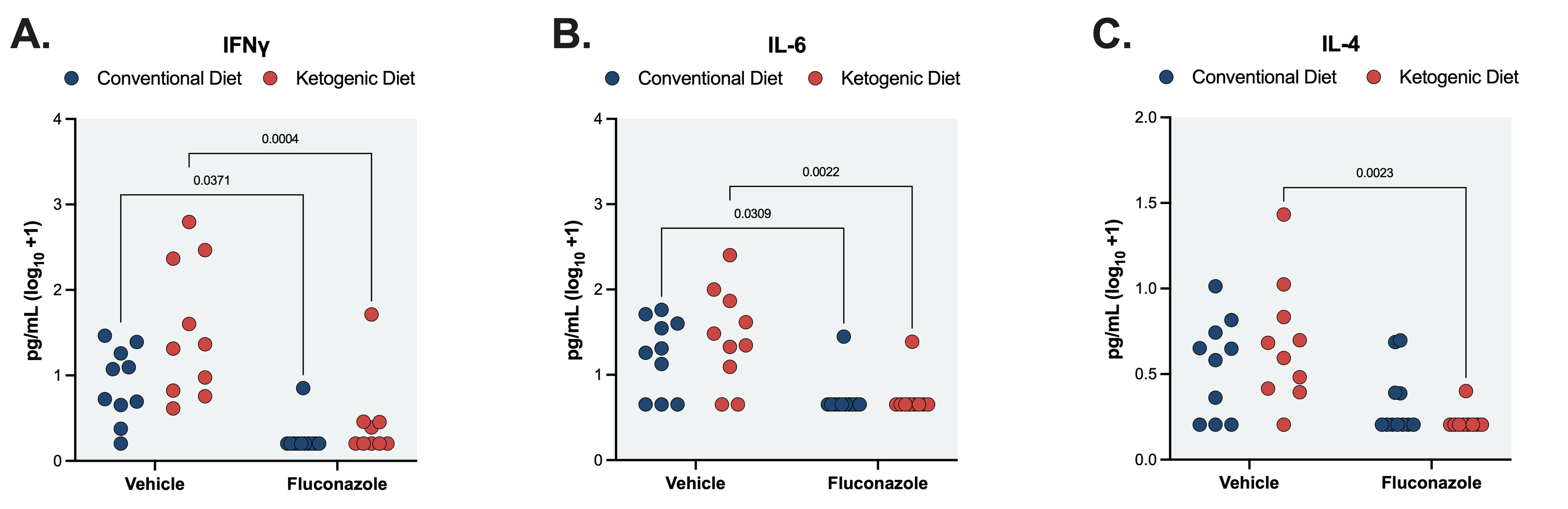

Supplement: Figure S5 — Serum cytokine profiles of mice infected with C. neoformans, collected 6 days post-infection, display some differences between vehicle and fluconazole-treated groups within each dietary cohort. [file mbio.00649-24-s0005.tiff]
