# Supplementary material for: A ketogenic diet enhances fluconazole efficacy in murine models of systemic fungal infection
Source: mBio. 2024 Apr 15;15(5):e00649-24. doi: 10.1128/mbio.00649-24 (PMC11077957; doi:10.1128/mbio.00649-24)
Supplement: Supplemental methods and legends — Additional experimental details and figure legends. [file mbio.00649-24-s0006.docx]

**Supplementary Materials**

**Measurement of fluconazole concentration in plasma**

Each plasma sample was diluted 1:10 in PBS in a 96-well plate. A calibration curve was generated in blank CD-1 mouse plasma (BioreclamationIVT) with fluconazole (Sigma) and d4-fluconazole (Cayman Chemical). One hundred microliters of each calibration solution, three quality control (QC) samples and two blanks, were also added to the 96-well plate. To each test well, 235 µL of an internal standard solution was added, and 235 µL of acetonitrile was added to the double blank. The plate was then shaken at 900 RPM for 5 minutes at room temperature on a Thermomixer (Eppendorf). Samples were chilled at -20 °C for 10 minutes and subsequently spun at 3000 rpm at 4 °C for 10 minutes. Then, 150 µL of each sample was transferred to a separate 96-well plate and 60 µL of water added to each well (final 50/50 v/v water/acetonitrile). Two study pool QCs were created by taking out an equal volume from all samples from each type and adding it to a blank well for each in the 96-well plate.

The analytical LC-MS/MS method, using selected reaction monitoring, was performed on an Acquity UPLC coupled to a Xevo TQ-S mass spectrometer using 5 µL injections. Mobile phase A consisted of 5 mM ammonium acetate containing 0.2% formic acid (FA) and 0.025% heptafluorobutyric acid in water, while mobile phase B was acetonitrile with 0.2% FA. A 2.1 mm x 50 mm BEH Shield RP18 column (Waters) with a flow rate of 0.50 mL/min and column temperature of 35 °C was utilized. The gradient was as follows: 1% B at 0 min, held at 1% B for one minute, 99% B at 2.0 min, held at 99% B for one-half minute, 1% B at 2.55 min, and held at 1% B until 4 minutes. The electrospray source conditions include 2.5 kV, 400 °C desolvation temperature, 1000 L/hr desolvation gas flow, 150 L/hr cone gas, and 7.0 bar nebulizer gas. MS/MS transitions for the most intense product ions were selected from an infusion experiment, and collision energy optimization was performed using LC-MS/MS and Skyline v20.2.1.454

**Measurement of fluconazole concentration in brain tissue**

Mouse brain tissue was homogenized with 0.7 mL 50/50 methanol/water on a Precellys 24 bead blaster (Bertin Instruments) at 4 °C for 3 cycles of 10 seconds at 10,000 rpm, with 30-second pauses in between each cycle. Homogenates were then centrifuged at 10 °C for 10 minutes at 4,000 rpm. Ten microliters of homogenate was added to a 1 mL 96-well NUNC plate along with 30 µL of 20 ng/mL fluconazole-d4 (Cayman Chemical) in acetonitrile and 60 µL 70/30 acetonitrile/water, and subsequently vortexed at 1,100 rpm for 15 minutes and centrifuged for 3 minutes at 3,000 rpm. The calibration curve was generated in naïve mouse brain homogenate.

Homogenate samples were analyzed with Waters Xevo TQ-S mass spectrometer. The separation was performed using a Waters 2.1 mm x 50 mm BEH Shield RP18 column. The flow rate was 0.5 mL/min with column temperature of 35 °C. The autosampler was set at 6 °C with 5 μL injection volume. Mobile phase A consisted of 5 mM ammonium acetate containing 0.2% FA and 0.025% heptafluorobutyric acid in water, and mobile phase B is acetonitrile with 0.2% FA. The linear gradient was as follows: 0-1 min, 1% B, 2-4 min, 99% B, 4.1-5.5 min, 1% B. The electrospray ionization source conditions include 2.5 kV high voltage, 400 °C desolvation temperature, 1000 L/hr desolvation gas flow, 150 L/hr cone gas, and 7.0 bar nebulizer gas. All data were analyzed in software Skyline v22.2. Fluconazole was quantified by multiple reaction monitoring with peak integration and the linear regression fit with 1/x weighting for the calibration curves: m/z 307.11 🡪 m/z 219.8 (calibration range of 20 – 2,000 ng/mL) and m/z 307.11 🡪 m/z 219.9 (calibration range of 80 – 40,000 ng/mL).

***MIC and FICs of ketone bodies***

To determine if ketone bodies demonstrated inherent antifungal activity, minimum inhibitory concentrations for β-hydroxybutyrate (BHB) (Sigma) and sodium butyrate (SB) (Thermo Scientific) were established for *C. neoformans* strain H99 in accordance with Clinical and Laboratory Standards Institute (CLSI) broth microdilution guidelines, M27-A3. Plates were incubated at 35 °C, and 50% growth inhibition at 48-hours was evaluated visually and spectrophotometrically at 530 nm.

Checkerboard assay of fluconazole and butyric acid

Checkerboard assays were performed with SB in combination with fluconazole against *C. neoformans* strain H99. Compound test concentrations were determined based on MIC values. Plates were incubated at 35 °C and combinatorial growth inhibition was determined visually and spectrophotometrically at 530 nm after 48-hours. At least three independent checkerboard assays were performed. The fractional inhibitory concentration index was calculated as previously described, and a FICI value of <0.5 indicated synergy [1].

**Supplementary References**

1. Steinbach WJ, Schell WA, Blankenship JR, Onyewu C, Heitman J, Perfect JR. In vitro interactions between antifungals and immunosuppressants against Aspergillus fumigatus. Antimicrob Agents Chemother. 2004;48(5):1664-9. doi: 10.1128/aac.48.5.1664-1669.2004. PubMed PMID: 15105118; PubMed Central PMCID: PMCPMC400573.

**Sup 1.** Mice have lowered glucose levels and reach a state of ketosis prior to and throughout infection with *C. neoformans* (*top*) and *C. albicans* (*bottom*) on a KD. Before dietary induction, on day 10 pre-inoculation, no differences were seen in blood ketone or glucose levels among all groups. **A.** After 9 days, KD mice had significantly higher ketone levels compared to CD (vehicle: P<0.0001, fluconazole: P=0.0016). Differences between KD and CD groups persist through 6 days post-inoculation with *C. neoformans* (vehicle: P=0.0024, fluconazole: P<0.0001). **B.** Blood glucose levels were lowered in KD mice after 9 days on a KD (vehicle: P=0.0019). On day 6 post-inoculation with *C. neoformans*, glucose levels were significantly lower in KD groups compared to CD (vehicle: P=0.0034, fluconazole: p=0.0002). **C.** Ketone levels were significantly increased in KD mice compared to CD after 9 days on a KD and throughout *C. albicans* infection (vehicle: P=0.0003, fluconazole: P=0.0001). **D.** After 9 days on a KD, blood glucose levels were significantly decreased in KD mice compared to CD (vehicle: P=0.0001, fluconazole: P=0.0003). After 7 days of infection with *C. albicans*, the difference in glucose levels across dietary groups was eliminated. Fluconazole KD mice had slightly elevated glucose levels compared to vehicle KD mice (P=0.0256). Mean ± SD of n = 5 mice per group. Panels top and bottom panels represent independent experiments. Mixed effects model with Geisser-Greenhouse correction and post-hoc Tukey test.

**Sup 2.** There are several differences in complete blood cell counts (*top*) and blood chemistry (*bottom*) between KD and CD-fed mice**. A.** White blood cell (WBC) counts were lower in vehicle- and fluconazole-treated KD mice compared to CD (P=0.0196 and 0.0028, respectively). Neutrophils were significantly lower in the KD fluconazole-treated group compared to CD (P=0.0294). Monocyte counts are significantly decreased in the vehicle-treated KD group compared to a CD (P=0.0087). Platelets were significantly lower in fluconazole-treated KD group versus CD (P=0.0006). **B.** Alkaline phosphatase **(**ALP) was lower in vehicle-treated infected KD mice compared to uninfected KD mice (P<0.001). Alanine transaminase (ALT) levels were significantly increased between KD and CD cohorts for the uninfected (P=0.0241) and fluconazole-treated (P=0.0078) groups. Phosphorus and lactate dehydrogenase (LDH) displayed differences only within the CD cohort. Mean ± SD of n ≥ 5 mice per group for top panel and n ≥ 3 for the bottom panel from a single experiment. Two-way ANOVA with post-hoc Tukey test.

**Sup 3.** The effect of a KD in combination with fluconazole is rapidly lost upon reversion to a CD. In combination with fluconazole, mice given a KD 10 days before infection and then switched to a CD at 8-hours post-inoculation displayed no difference in mean brain fungal burden than mice receiving a CD for the duration of the study. Mice beginning a KD at 8-hours post-inoculation had statistically equivalent brain fungal burden as mice started on a KD 10 days prior to infection with fluconazole treatment. Mean ± SEM of n ≥ 5 mice per group from one experiment. One-way ANOVA with post-hoc Tukey test.

**Sup 4.** While sodium butyrate synergized with fluconazole *in vitro*, its antifungal effects were unable to be replicated *in vivo.* **A.** Mice treated with 1.2 g/kg SB, i.p., displayed no difference in brain fungal burden compared to vehicle-treated controls. Combination treatment of SB (at 1.2, 0.8, and 0.4 g/kg, i.p.) and 80 mg/kg fluconazole, i.p., displayed no greater decrease in fungal burden compared to fluconazole treatment, alone. **B.** Mice administered 100 mM SB in drinking water displayed no decrease in mean brain fungal burden compared to untreated controls. Mice receiving a combination of 100 mM SB and 80 mg/kg fluconazole, i.p., showed no greater reduction of brain fungal burden compared to fluconazole monotherapy. Mean ± SEM of n = 5 mice per group for panel A and n = 4 mice per control group and n ≥ 9 mice per SB treatment group in panel B. Panels A and B represent two independent experiments. One-way ANOVA with post-hoc Tukey test.

**Sup 5.** Serum cytokine profiles of mice infected with *C. neoformans,* collected 6 days post-infection display some differences between vehicle and fluconazole-treated groups within each dietary cohort. **A**. IFNy expression was decreased upon fluconazole treatment on both a CD (P=0.0371), and a KD (P=0.0004). **B.** IL-6 expression was lowered upon fluconazole treatment for both KD (P=0.0022) and CD (P=0.0309) mice, compared to vehicle controls. **C**. Fluconazole-treated KD mice had lower IL-4 expression compared to vehicle-treated mice (P=0.0023). Cytokine concentrations from at least 9 mice per group were (log_10_ + 1) transformed. Two-way ANOVA with post-hoc Tukey test.
